# Proud Syndrome: A Rare Cause of Corpus Callosum Agenesis

**DOI:** 10.7759/cureus.40671

**Published:** 2023-06-20

**Authors:** Risha Devi, Suman Chaurasia, Mayank Priyadarshi, Poonam Singh, Sriparna Basu

**Affiliations:** 1 Neonatology, All India Institute of Medical Sciences, Rishikesh, Rishikesh, IND

**Keywords:** x-linked inheritance, neonate, arx gene, proud syndrome, corpus callosum agenesis

## Abstract

Agenesis of the corpus callosum (ACC) is one of the most common congenital brain anomalies with variable associations and outcomes. The incidence of ACC varies from 1.8 per 10,000 live births in normal children to as high as 600 per 10,000 in children with neurodevelopmental problems. Here, we report the case of a female neonate delivered in our institute at term gestation to a gravida 4 mother with partial ACC. The neonate was antenatally diagnosed with ACC. The mother had a previous fetus with a supratentorial cyst that was medically terminated. The neonate had a normal clinical examination, but the ultrasound of the cranium suggested ACC. Given the significant family history, a clinical exome sequencing test revealed a pathogenic frameshift mutation in the *ARX *gene that causes Proud syndrome. We discuss the relevant points in the diagnosis, workup, and prognosis of ACC through this case. This case highlights the importance of antenatal assessment for timely amniocentesis and a genetic diagnosis to guide the parental decision for continuation of the pregnancy, level 2 scans to detect associated anomalies, and postnatal assessment to determine the cause and prognosis of a neonate with ACC.

## Introduction

The corpus callosum is the largest connective tissue in the human brain composed of approximately 190 million neurons, with the primary function being an exchange of information between cerebral hemispheres [1]. Agenesis of the corpus callosum (ACC) is a common congenital brain anomaly that is usually detected antenatally. Its prevalence in a Western cohort has been documented to be 1.8 per 10,000 live births which increases up to 600 per 10,000 on assessment of children with neurodevelopmental problems [2,3]. ACC includes both partial or complete absence of the corpus callosum depending on the timing of insult during the antenatal period [4]. Clinical presentation is varied depending on the chromosomal, neurological, and clinical associations in individual patients [5]. Here, we report a rare chromosomal association in a neonate with ACC and discuss the varying outcomes in neonates with ACC.

## Case presentation

A 29-year-old, gravida 4, booked mother delivered a female baby vaginally at 40 weeks and one day of gestation at our institute. The mother’s first pregnancy was medically terminated at 20 weeks of gestation in view of a large para-midline anechoic supratentorial cyst in the fetus. Her second pregnancy resulted in a live healthy male baby, while the third pregnancy resulted in a blighted ovum followed by an abortion at one and a half months of amenorrhea. The current pregnancy was booked, and on a level 2 scan, the fetus was found to have an anechoic lesion in the brain which was investigated further with a fetal MRI. MRI revealed isolated ACC. She did not have a history suggestive of an intrauterine infection, and her TORCH screening was negative. The mother’s blood group was O Rh-negative, not iso-immunized, and she received anti-D immunoglobulin. The rest of the antenatal period was uneventful, and all antenatal visits were completed. The mother had partial ACC, with a history of seizure disorder controlled on a single antiepileptic between 1996 and 2008 with no requirement of any antiepileptic for the last 13 years.

**Figure 1:**
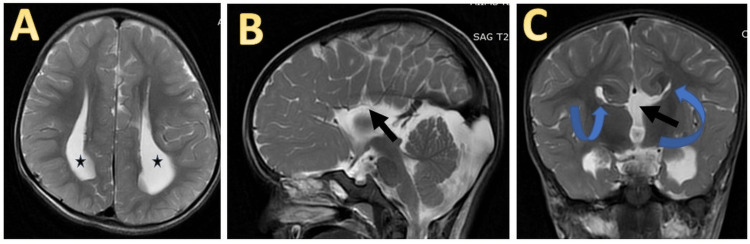
(A) T2-weighted MRI showing the widely separated body of the lateral ventricle with parallel orientation (*). (B) T2-weighted sagittal MRI showing a complete absence of the corpus callosum and cingulate sulcus with the hemispheric sulci seen reaching the third ventricle in a radial fashion giving a spoke wheel appearance (arrow). (C) T2-weighted coronal MRI showing a high-riding third ventricle (straight black arrow) communicating with interhemispheric cistern and the Viking helmet appearance of lateral ventricles (curved arrow).

The neonate cried immediately after birth. The APGAR score was 8 and 9 at one and five minutes, respectively. The neonate received routine care at birth. The birth weight was 2,940 g which was appropriate for gestational age per the Intergrowth-21 newborn size at birth charts. Length and head circumference was 48 cm and 33.5 cm, respectively, both between the 10th and 50th centile per the Intergrowth-21 size at birth charts. General physical and neurological examinations at birth were normal, and the neonate was initiated on breastfeeding. A detailed workup for antenatally diagnosed ACC was done. An ophthalmological examination was done to rule out chorioretinal lacunae without any significant findings. Her TORCH screening was negative. Ultrasound of the cranium revealed an absence of the corpus callosum, colpocephaly, and a subependymal cyst in the left caudothalamic groove without any features of hydrocephalus. Ultrasound of the abdomen, kidney, and urinary bladder was normal. Echocardiography revealed a 5 mm atrial septal defect. Automated brainstem evoked response audiometry was normal bilaterally and so was the infantogram. MRI revealed an absence of the corpus callosum with colpocephaly and a characteristic *Viking helmet* appearance (Figure 1).

Clinical exome sequencing was done on the advice of a medical genetics specialist and revealed a frameshift mutation in the *ARX *gene suggestive of Proud syndrome (Table 1). The neonate had an uneventful hospital stay, was feeding well, did not have seizures, and was discharged at six days of life. Neurological examination at discharge was normal. The neonate has been under follow-up and is doing well. The neurological examination at three, six, and nine months of age has been normal, and the neonate has attained all the expected developmental milestones per the Denver development screening test II.

**Table 1:** Mutations detected on clinical exome sequencing.

Gene and transcript	Variant	Location	Zygosity	Disorder (OMIM)	Inheritance	Classification
*RBM20 NM 001134363.3*	c.1210G>T (p.Gly404Cys)	Exon 2	Heterozygous	Dilated cardiomyopathy 1DD (613172)	Autosomal dominant	Uncertain significance
*ARX NM_139058.3*	c.1013_1019dupACCAGCT (p.Glu341Prots•193)	Exon 2	Heterozygous	Proud syndrome (300004)	X-linked	Likely pathogenic

## Discussion

The development of the corpus callosum is a complex process involving midline patterning, telencephalic hemisphere formation, neuronal specification, and guidance of their axons [6]. This development is affected by a plethora of genetic, infectious, vascular, and environmental causes. Observational studies report that, overall, 30-45% of ACC cases have an identifiable cause (10% chromosomal anomalies, 20-35% known genetic syndromes), while in only 25% of cases of complete ACC, a cause can be identified [7]. Mutations in the *ARX*, *L1CAM*, *ZHFX1B*, *Dcc*, *Gap43*, *MRPS16*, *KCC3*, and *SPG11* genes, as well as syndromes such as Andermann, Mowat-Wilson, Proud, Aicardi, Acrocallosal, and Vici syndrome, have been reported to result in ACC [8]. Other causes include fetal alcohol syndrome, hypothyroidism, intrauterine infections, metabolic causes such as pyruvate dehydrogenase deficiency, neonatal adrenoleukodystrophy, maternal phenylketonuria, and, rarely, vascular insults [4].

Antenatal diagnosis of ACC depends on a scan done later than 18 weeks, and MRI is needed to detect associated neurological abnormalities. Amniocentesis for chromosomal analysis or microarray is usually advised to detect syndromes that would guide parental decisions regarding the continuation of pregnancy. Postnatally, a detailed history and clinical and radiological assessment should be done to look for other abnormalities and further workup based on these findings. These modalities may include MRI, next-generation sequencing, and tests for metabolic disorders or intrauterine infections [4]. It has been reported that up to 15% of neonates diagnosed to have isolated ACC antenatally have associated abnormalities detected after birth [9]. Outcomes associated with ACC are extremely variable and might not be similar even in patients with identical neuroimaging findings [4]. A recent meta-analysis reported that 76% of neonates diagnosed with isolated ACC had a normal neurodevelopmental outcome while 8% had severe disabilities. Cognitive and language domain was affected in 15% and 8% of cases, respectively.

In 1992, Proud and co-workers reported features of a new syndrome in a family over four generations wherein the male members were more severely affected compared to the females [10]. Associated features reported were ACC, acquired micrencephaly, seizures, mental retardation, limb contractures, kyphoscoliosis, long tapering fingers with hyperconvex nails, synopsis, optic atrophy, and urologic abnormalities such as renal dysplasia, cryptorchidism, and hypospadias. Spastic quadriplegia, emotional lability, and clinically normal carrier state were reported in the females. The authors used DNA linkage analysis to conclude that the region was between Xp11.3-21.3.

Later Kato and co-workers reviewed clinical and genetic data from 29 males and multiple females from 16 families and found that *ARX* gene mutations (Xp21.3) of varying severity led to a spectrum of neurological disorders, including hydranencephaly, lissencephaly, infantile spasms, dystonias, mental retardation, and isolated ACC which might be associated with ambiguous genitalia to a completely normal brain structure development associated with other syndromes [11].

The case reported by us is of a female neonate with no clinical or radiological abnormalities associated with ACC. A clinical exome sequencing was done in view of a previous fetus having a supratentorial cyst and the mother having partial ACC. A three-generation pedigree of the family was otherwise normal. This test helped us confirm the diagnosis and prognosticate parents regarding outcomes in this neonate and in any future pregnancy.

## Conclusions

ACC is a neurological anomaly that has varying manifestations depending upon its syndromic and non-syndromic associations. A detailed workup should be done in each case of ACC to detect associated anomalies and prognosticate parents regarding subsequent neurodevelopmental outcomes and follow-up. Next-generation sequencing methods would prove to be potent tools for the identification of rare syndromes in isolated ACC and the prognostication of these cases.
